# Changes in tobacco and e-cigarette consumption in Spanish university students during the COVID-19 lockdown: Identification of vulnerable groups

**DOI:** 10.18332/tid/156109

**Published:** 2023-01-11

**Authors:** Víctor J. Villanueva-Blasco, Sergio Veiga Rodeiro, Verónica Villanueva-Silvestre, Manuel Isorna Folgar, Miriam Otero Requeijo, Andrea Vázquez-Martínez, Vicente Zanón-Moreno, Adelaida Lozano-Polo

**Affiliations:** 1 Faculty of Health Sciences, Valencian International University, Valencia, Spain; 2 Health and Psycho-Social Adjustment Research Group, Valencian International University, Valencia, Spain; 3 Faculty of Education and Social Work, University of Vigo, Vigo, Spain; 4 Faculty of Medicine, University of Murcia, Murcia, Spain

**Keywords:** tobacco, age, gender, university healthy campuses, living situation

## Abstract

**INTRODUCTION:**

The objective of this study was to determine the changes in tobacco consumption in Spanish university students during the lockdown in 2020, and to identify vulnerable groups based on gender, age, and living situation.

**METHODS:**

This was a cross-sectional descriptive study. From a sample of 1540 Spanish university students, 19.9% (n=306; 77.6% women; mean age=30.9 years; SD=8.3) reported having consumed tobacco. The frequency and average daily consumption of cigarettes and electronic nicotine delivery systems (ENDS) before the pandemic and during lockdown were analyzed.

**RESULTS:**

A total of 97.1% of students consumed only cigarettes, 2.9% only ENDS, and 3.3% were dual consumers. During lockdown, cigarette consumption was significantly reduced (5.3 before; 4.0 during; t_(71)_=3.6255; p<0.001) in the youngest group (aged 18–24 years). However, women daily users significantly increased their consumption (t_(149)_= -2.5461; p<0.05) and so did the 35–44 years age group (t_(32)_= -2.2285; p<0.05). Cigarette consumption significantly increased in those who were living alone (5.6 to 7.2; Z= -2.351; p<0.05) and with a partner (7.2 to 8.0; t_(97)_= -2.3771; p<0.05), but decreased in those who were living with their parents or other relatives (6.2 to 4.5; t_(101)_=3.4298; p<0.001). A total of 17.0% ceased consumption during lockdown, mainly women, younger students (aged 18–24 years), and those who lived with their parents. None of the people who used cigarettes daily during the pre-pandemic period stopped smoking during lockdown.

**CONCLUSIONS:**

Younger university students and those living with their parents decreased their tobacco use during the lockdown. Potentially vulnerable groups at risk of increasing their consumption were women who consumed tobacco daily before the pandemic and students aged 35–44 years who lived alone or with their partner.

## INTRODUCTION

Healthy campuses^1,2^ are an example of how universities can be organized as health promoting agents^3^ through their structures. Tobacco control policies aimed at tobacco-free campuses are effective in reducing on-site consumption^4,5^ and are increasing in popularity over time^5-7^. Likewise, the training of health-promoting agents among university students, who subsequently participate in awareness-raising and information activities, has also shown promising results^8^.

To contain the COVID-19 pandemic, universities had to follow measures, including cancelling face-to-face teaching and transferring it to an online format^9^. In some countries, teaching the core part of the curriculum became the priority, to the detriment of other actions such as promoting healthy habits. This meant bringing all health promotion actions carried out in the university environment to a halt, which negatively impacted the efforts developed against the tobacco epidemic^10^. These measures, including social distancing and lockdowns, have had social and economic consequences that may have increased tobacco use and smoking relapse^11^.

Studies focused on the lockdown period in the university context are still scarce. In terms of tobacco use, some have found significant reductions in the amount and frequency of tobacco and e-cigarette (electronic nicotine delivery systems, ENDS) use during lockdown^12-14^. However, they attribute this to the closure of campuses and students not being able to get together and engage in social events due to social isolation measures. This same phenomenon has been observed in the alcohol consumption of young people aged 18–29 years and in those who lived with their parents, with alcohol consumption decreasing significantly during lockdown^15^. However, a 58% increase has also been observed in male students who smoked at least one cigarette per day^12^. In some studies, these increases have been associated with psychological distress linked to pandemic circumstances^16^.

At present, universities are trying to resume their health promotion actions and identify vulnerable groups in the strategic planning of these healthy campuses. However, the few existing studies do not identify vulnerable groups, nor do they contemplate the heterogeneity of university students that brings together people of different age ranges, some of whom balance their studies with professional work or family^17^. An individual’s living situation is a social variable commonly used in epidemiological surveys because of its impact on health. The return of the youngest students to the family home during lockdown could have been a relevant contextual prevention measure; those who lived alone could have been exposed to greater psychological stress during the lockdown and increased their consumption. Likewise, students who had to combine studies, work, and family could be exposed to greater stressors that increased their tobacco consumption.

This study aimed to determine changes in tobacco consumption patterns in university students during the COVID-19 lockdown period compared to the previous pre-pandemic period. In addition, it sought to establish groups who increased their tobacco use and, therefore, their risk of addiction during this period, and groups that ceased their consumption. The changes were analyzed according to gender, age, and living situation. We expected to find a decrease in tobacco consumption among the youngest university students but increases among those aged >30 years, given that their socio-family and contextual consumption characteristics may differ.

## METHODS

This was a non-probabilistic descriptive study with convenience sampling. The sample of the reference study that analyzed the effects of the lockdown on a set of sociodemographic, health, and addictive behavior variables was composed of 3780 people aged 18–64 years, residents of the 17 Spanish autonomous communities and two Spanish autonomous cities. After applying the inclusion criteria, the sample for this study consisted of 306 people who reported being enrolled in university studies and had used tobacco in the six months prior to the survey. A total of 77.6% of these 306 tobacco-using participants were women. The overall mean age was 30.9 years (SD=8.3), 30.9 years (SD=8.5) for women and 31.1 years (SD=7.8) for men. The breakdown in age was: 24.5% (18–24 years), 33.7% (25–29 years), 17.7% (30–34 years), 17.0% (35–44 years), 5.6% (45–54 years), and 1.6% (55–64 years).

Concerning the living situation, 38.5% reported that at the time of the study, they lived with their parents or other relatives, 35.6% with their partner, 8.6% in family units with under-aged children, 8.3% alone, 8.3% shared a flat, and 0.7% had other types of living situations. A total of 76.6% of the sample reported that their living situation had not changed compared to the pre-pandemic period. Of the remaining 23.4% that reported that their living situation had changed as a result of the lockdown, 57.5% went to live with their parents or other relatives, 21.7% went to live with their partner, 8.1% went to live with children under 16 years of age, and 5.6% started living alone.

### Procedure

A battery of instruments about smoking habits was applied in an online questionnaire, considering the coverage error for online surveys, reducing it by establishing age ranges of the study population with adequate internet access according to the Survey on Equipment and Use of Information and Communication Technologies in Households^18^. Data collection began on 14 April 2020, 30 days after the start of the first and the exceptional lockdown measures in Spain under the Royal Decree of 14 March 2020, declaring the state of alarm for COVID-19^19^, and ended on 29 May, with the start of the de-escalation period. Collaboration requests were made using the ‘snowball’ technique through web hosting, social networks, email, and telephone messaging applications, which are especially relevant in the university environment. Participants were informed of the voluntary nature of their participation in accordance with the Organic Law 3/2018 on the Protection of Personal Data and Guarantee of Digital Rights^20^. The inclusion criteria were: a) aged 18–64 years, according to the age ranges established by the epidemiological report ‘Survey on Alcohol and Drugs in Spain (EDADES)’^21^; b) accepted to participate in the study; and c) enrolled in a university. The exclusion criteria were: missing values, or inconsistent response patterns.

### Study variables

Sociodemographic variables were collected: gender (men, women); age, according to the groups established in the EDADES report^20^ (18–24, 25–29, 30–34, 35–44, 45–54, 55–64 years); and living situation (living alone during lockdown; living with parents or other relatives during lockdown; living with a partner during lockdown; sharing a flat with people who are not relatives or a partner during lockdown; living with children aged ≤16 years during lockdown; another living situation).

To analyze tobacco consumption and obtain a representative consumption frequency for the pre-pandemic period, the participants were questioned about their average days of consumption per month during the last six months, offering response alternatives considered in the EDADES survey^22^ for consumption in the previous 30 days: 0) None; 1) 1–2 days; 2) 3–4 days; 4) 5–9 days; 5) 10–19 days; and 6) ≥20 days. In addition, consumption in the last seven days (from 0 to 7 days) was requested to establish the frequency of weekly consumption during lockdown to establish daily consumption. In both cases, questions were asked about tobacco/nicotine consumption in the form of cigarettes and ENDS. In addition, the average daily consumption before the pandemic and during the lockdown, regarding cigarettes and ENDS, was also requested by asking for the number of cigarettes consumed on average for both periods.

### Statistical analysis

The statistical analysis was performed with Stata, version 17^23^. A correction of 0.5 years was applied to the age variable to reduce the bias derived from registering the age of the participants in completed time instead of elapsed time. A descriptive analysis of the sociodemographic variables and the consumption of cigarettes and ENDS was conducted. Tobacco users who reported using both cigarettes and ENDS were termed, dual users. Student’s t-tests or Wilcoxon’s signed-rank tests were applied to analyze the evolution of the average number of cigarettes consumed between the pre-pandemic and lockdown periods (when the requirements to use the parametric test were not met), checking the assumptions of normality (Shapiro-Wilk) and homoscedasticity (Levene). The comparison was made between those who reported consumption in the previous six months in the complete sample based on gender, age group, and living situation. In the case of ENDS, due to the small sample size, the comparison was only made for the complete sample and those who reported consumption in the previous six months. Cohen’s d was used to estimate the magnitude of the effect of the differences.

To analyze the intragroup differences in average consumption, both before and during the lockdown, depending on the age groups and the living situation, various analyses of variance (ANOVA and Kruskal-Wallis) were conducted. Contrasts with Bonferroni correction to assess the differences between pairs were carried out. Concerning smoking cessation during the lockdown, a descriptive analysis was carried out according to age group and living situation.

The evolution of cigarette consumption before the pandemic and during the lockdown was analyzed for the subsample of university students aged <25 years, considering the changes in their living situation. Wilcoxon’s signed-rank test was applied to analyze the evolution of the average number of cigarettes consumed between the pre-pandemic and lockdown periods. An analysis of variance was conducted to analyze the intragroup differences in average consumption, both before and during the lockdown. In all the statistical tests, significant values were those with a p<0.05.

## RESULTS

Regarding tobacco consumption, 19.9% (n=306) reported having consumed in the 6 months prior to the survey (20.2% women; 18.9% men), with 97.1% (n=297) of them having smoked cigarettes and 6.2% (n=19) having used ENDS. Of this cigarette and ENDS users, 3.3% (n=10) reported dual use of cigarettes and ENDS. Given the small sample size of this subgroup, and to simplify the analyses, these ten dual consumers were considered in subsequent analyses by including them both in the subsample of cigarette consumers and the subsample of ENDS users.

In terms of the average frequency of monthly consumption in the last 6 months: 67.7% (n=207) of the participants reported consuming ≥20 days; 8.5% (n=26) 10–19 days; 8.8% (n=27) 5–9 days; 6.5% (n=20) 3–4 days; 5.9% (n=18) 1–2 days; and 2.6% (n=8) <1 day. Regarding daily average cigarette consumption in the previous 7 days during lockdown: 63.4% (n=194) consumed every day; 6.2% (n=19) 4–6 days; 11.4% (n=35) 1–3 days; and 19.0% (n=58) with a frequency of <1 day.

**Figure 1:**
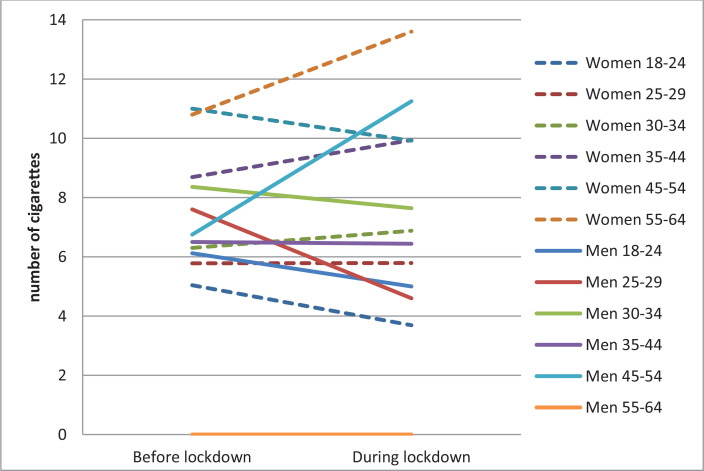
Average number of tobacco cigarettes consumed per day before the pandemic and during the COVID-19 lockdown according to gender and age in Spanish university students, 2020 (N=297)

**Figure 2:**
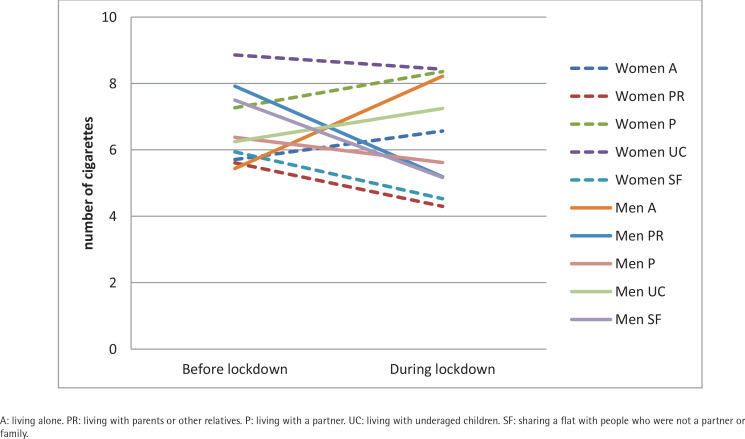
Average number of tobacco cigarettes consumed per day before the pandemic and during the COVID-19 lockdown according to gender and living situation in Spanish university students, 2020 (N=268)

The analysis of the evolution of average daily tobacco consumption in the last six months and during lockdown, revealed no statistically significant differences between consumers regarding the number of cigarettes or ENDS nor the number of cigarettes according to gender. However, depending on the age, a statistically significant reduction is observed in the youngest group (5.3 before; 4.0 during; t_(71)_=3.6255; p<0.001) (Table 1 and Figure 1). The analysis based on the living situation during lockdown (Table 1 and Figure 2) revealed significant increases in those who were living alone (5.6 to 7.2 cigarettes/day) (Z= -2.351; p<0.05) and those living with a partner (7.2 to 8.0 cigarettes/day; t_(97)_=2.3771; p<0.05). On the other hand, consumption decreased significantly in those who were living with their parents or relatives (6.2 to 4.5 cigarettes/day; t_(101)_=3.4298; p<0.001). Statistically significant differences were found in average consumption according to the living situation during lockdown (χ^2^=21.383; p<0.001).

**Table 1:** Evolution of the average daily consumption of conventional cigarettes and electronic cigarettes (ENDS) before the pandemic and during lockdown in Spanish university students, 2020 (N=297)

	*n*	*Before lockdown Mean (SD)*	*During lockdown Mean (SD)*	*Diff.*	*t/Z*	*p*	*d*
**E-cigarettes**	19	7.1 (7.2)	5.2 (5.9)	-1.9	0.1210	0.9172	
**Cigarettes**							
**All**	297	6.7 (5.8)	6.4 (6.5)	-0.3	1.0016	0.3174	
**Sex**							
Women	229	6.5 (5.6)	6.5 (6.6)	0	0.1004	0.9201	
Men	68	7.0 (6.3)	6.0 (6.4)	-1.0	1.5049	0.1371	
**Age** (years)							
18–24	72	5.3 (4.3)	4.0 (5.1)	–0.7	3.6255	0.0005	0.43
25–29	101	6.1 (5.2)	5.6 (5.3)	-0.5	1.2283	0.2222	
30–34	51	6.7 (5.5)	7.0 (5.9)	0.3	-0.4578	0.6491	
35–44	51	8.0 (7.5)	8.8 (8.3)	0.8	-1.3837	0.1726	
45–54	17	10.0 (6.2)	10.2 (7.8)	0.2	-0.1480	0.9023	
55–64	5	10.8 (8.4)	13.6 (10.4)	2.8	-0.8160	0.5000	
**LS**							
LA	23	5.6 (5.3)	7.2 (7.8)	1.6	-2.351	0.0170	0.37
LF	102	6.2 (5.4)	4.5 (5.3)	-1.7	3.4298	0.0009	-0.34
LP	98	7.2 (6.5)	8.0 (6.7)	0.8	-2.3771	0.0194	0.24
LC	22	7.9 (6.4)	8.0 (8.0)	0.1	-0.1960	0.8527	
SF	23	6.4 (4.5)	4.7 (4.7)	-1.7	1.8590	0.0650	

LS: living situation during lockdown. LA: living alone. LF: living with parents or other relatives. LP: living with a partner. LC: living with children aged <16 years. SF: sharing a flat with people who were not a partner or family. t: Student’s t-test. Z: PRS Wilcoxon. p: significance level. d: Cohen’s d. Diff.: absolute difference between before and during lockdown.

Furthermore, the analysis of the differences between groups for each time period revealed no significant differences for daily consumption based on gender, before the pandemic (6.5 women and 7.0 men; t_(295)_= -0.6316; p=0.5281), nor during lockdown (6.5 women and 6.0 men) (t_(295)_=0.5553, p=0.5791). There were only statistically significant differences during lockdown according to age (χ^2^=27.498; p<0.001). Post hoc analyses indicated that the pre-pandemic consumption of the age group of 18–24 years was lower than the age group of 45–54 years (5.3 vs 10.0, respectively, p<0.05). During lockdown, the consumption according to age was: 4 for 18–24 years, 7.0 for 30–34 years, 8.8 for 35–44 years, and 10.2 for 45–54 years (all p<0.05). A posteriori contrasts indicated that during the lockdown, daily consumption of those who were living with parents or relatives was significantly lower than among those who were living with their partner (4.5 vs 8.0, respectively, p<0.0001).

Table 2 provides information on the evolution of daily cigarette consumption during lockdown compared to the pre-pandemic period. Around 39% of smokers decreased their consumption, and another similar percentage increased it, slightly higher in women (39.7%) than in men (33.8%). Considering age, an increase in daily cigarette consumption was observed as age increased, with that of the age group of 55–64 years (60%) being more than double that of those aged 18–24 years (26.4%). However, the increase observed in the 45–54 years age group was similar (29.4%) to that of the younger group which had the highest rate of consumption maintenance (41.2%).

**Table 2:** Changes in the average daily consumption of cigarettes during lockdown according to age group in Spanish university students, 2020 (N=297)

		*Total* *% (n)*	*Age (years)*					
			*18–24*	*25–29*	*30–34*	*35–44*	*45–54*	*55–64*
			*% (n)*	*% (n)*	*% (n)*	*% (n)*	*% (n)*	*% (n)*
**Total**	Decreased	38.7 (115)	56.9 (41)	39.6 (40)	29.4 (15)	25.5 (13)	29.4 (5)	20.0 (1)
	Maintained	22.9 (68)	16.7 (12)	20.8 (21)	23.5 (12)	29.4 (15)	41.2 (7)	20.0 (1)
	Increased	38.4 (114)	26.4 (19)	39.6 (40)	47.1 (24)	45.1 (23)	29.4 (5)	60.0 (3)
**Women**	Decreased	38.0 (87)	58.2 (32)	37.0 (30)	30.0 (12)	20.0 (7)	38.5 (5)	20.0 (1)
	Maintained	22.3 (51)	16.4 (9)	18.5 (15)	22.5 (9)	31.4 (11)	46.2 (6)	20.0 (1)
	Increased	39.7 (91)	25.5 (14)	44.4 (36)	47.5 (19)	48.6 (17)	15.4 (2)	60.0 (3)
**Men**	Decreased	41.2 (28)	52.9 (9)	50.0 (10)	27.3 (3)	37.5 (6)	- (0)	- (0)
	Maintained	25.0 (17)	17.7 (3)	30.0 (6)	27.3 (3)	25.0 (4)	25.0 (1)	- (0)
	Increased	33.8 (23)	29.4 (5)	20.0 (4)	45.5 (5)	37.5 (6)	75.0 (3)	- (0)

Considering both gender and age (Table 2 and Figure 1), the trend in women was the same as the pattern previously described for the sample set, with a small percentage of people aged 45–54 years increasing their daily consumption during lockdown (15.4%) compared to approximately three times the number of women maintaining it (46.2%). However, the trend in men differed. The percentage of men who increased their daily consumption in the age group of 45–54 years was 2.6 times higher (75%) than in the age group of 18–24 years (29.4%). However, this upward trend is not linear.

When analyzing the percentage of people who decreased, maintained, or increased their average daily cigarette consumption depending on their living situation during lockdown (Table 3 and Figure 2), the most significant reductions in consumption are seen in those who were living with their parents or other relatives (58.8%) and among those who were sharing a flat (47.8%). The greatest increases were observed in those who were living alone (60.9%), with a partner (52.0%), and living with under-aged children (40.9%). According to gender, significant increases in consumption are observed in both men and women living alone, although more in men (77.8%) than in women (50%). Among those living with a partner, daily consumption increased in 55.3% of women compared to 30.8% of men. In those living with under-aged children, women increased their consumption by 35.7% compared to 50% in men. Regarding those sharing a flat, the consumption increase in men was almost double (33.3%) that in women (17.7%). Among those who were living with parents or other relatives, both genders presented a similar decreased consumption of about 25%. However, more women (60.5%) than men (53.9%) reduced their consumption.

**Table 3:** Changes in daily average cigarette consumption during lockdown depending on living situation in Spanish university students, 2020 (N=268)

		*Total* *% (n)*	*Living situation during lockdown*				
			*Alone*	*With parents or relatives*	*With partner*	*With under-aged children*	*Sharing a flat*
			*% (n)*	*% (n)*	*% (n)*	*% (n)*	*% (n)*
**Total**	Decreased	38.8 (104)	17.4 (4)	58.8 (60)	21.4 (21)	36.4 (8)	47.8 (11)
	Maintained	22.0 (59)	21.7 (5)	15.7 (16)	26.5 (26)	22.7 (5)	30.4 (7)
	Increased	39.2 (105)	60.9 (14)	25.5 (26)	52.0 (51)	40.9 (9)	21.7 (5)
**Women**	Decreased	38.4 (79)	28.6 (4)	60.5 (46)	17.7 (15)	35.7 (5)	52.9 (9)
	Maintained	21.8 (45)	21.4 (3)	13.2 (10)	27.1 (23)	28.6 (4)	29.4 (5)
	Increased	39.8 (82)	50.0 (7)	26.3 (20)	55.3 (47)	35.7 (5)	17.7 (3)
**Men**	Decreased	40.3 (25)	- (0)	53.9 (14)	46.2 (6)	37.5 (3)	33.3 (2)
	Maintained	22.6 (14)	22.2 (2)	23.1 (6)	23.1 (3)	12.5 (1)	33.3 (2)
	Increased	37.1 (23)	77.8 (7)	23.1 (6)	30.8 (4)	50.0 (4)	33.3 (2)

**Figure 3:**
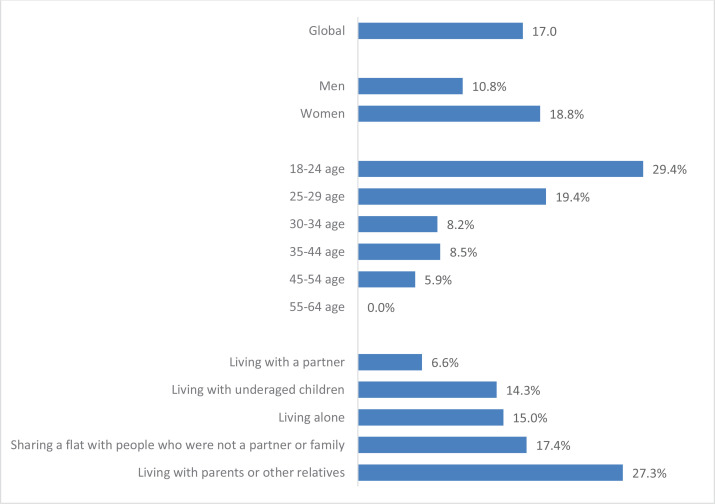
Smoking cessation rate during lockdown among those who had smoked cigarettes in the 6 months prior to the study, in Spanish university students, 2020 (N=268)

Concerning the data referring to smoking cessation during lockdown among those who had consumed cigarettes in the six months prior to the study (Figure 3), 17.0% (n=48) reported having quit smoking as a result of the lockdown, of which 85.4% were women (n=41). Based on age, 29.4% aged 18–24 years, 19.4% aged 25–29 years, 8.2% aged 30–34 years, and 5.9% aged 45–54 years quit smoking cigarettes, while none of those aged 55–64 years quit. According to the living situation during the lockdown, 15.0% of those who lived alone, 27.3% of those who lived with their parents or other relatives, 6.6% of those who lived with their partner, 14.3% of those who lived in family units with under-aged children, and 17.4% of those who share a flat reported having quit smoking.

Among those who reported having used cigarettes daily in the last seven days (Table 4), the average consumption increased significantly compared to the pre-pandemic situation. The analysis of the evolution by gender revealed a significant increase only in women (8.3 to 9.2 cigarettes; t_(149)_= -2.7493; p<0.05). Depending on age, consumption increased significantly only in the age group of 35–44 years (10.9 to 12.7 cigarettes; t_(32)_= -2.2285; p<0.05). A statistically significant increase was observed among those who lived alone (9.4 to 12.3) and those who lived with their partner (8.5 to 9.8). There were no significant differences according to gender before the pandemic (8.3 women and 8.8 men; t_(186)_= -0.5100; p=0.6107) or during lockdown (9.2 women and 9.1 men; t_(186)_=0.0918; p=0.9270). Significant differences in average daily consumption according to age were observed in the lockdown period (χ^2^=20.681; p<0.001). The post hoc contrasts indicated that the cigarettes/day consumption of people aged 18–24 years was lower than that of people aged 35–44 years (6.8 vs 12.7, respectively; p<0.05) during the lockdown. However, no differences were observed in the average daily consumption before or during lockdown according to the living situation. Regarding smoking cessation, none of the people who consumed cigarettes daily during the pre-pandemic period reported having stopped their consumption during the lockdown.

**Table 4:** Evolution of average daily cigarette consumption before the pandemic and during lockdown among those who reported daily consumption in the last 7 days in Spanish university students, 2020 (N=188)

	*n*	*Before lockdown Mean (SD)*	*During lockdown Mean (SD)*	*Diff.*	*t/Z*	*p*	*d*
**All**	188	8.4 (5.8)	9.2 (6.5)	0.8	-2.5461	0.0117	0.19
**Sex**							
Women	150	8.3 (5.8)	9.2 (6.4)	0.9	-2.7493	0.0067	0.22
Men	38	8.8 (5.6)	9.1 (6.7)	0.3	-0.3431	0.7335	
**Age** (years)							
18–24	39	7.6 (4.3)	6.8 (5.5)	-0.8	1.6567	0.1058	
25–29	66	7.1 (4.7)	7.9 (4.8)	0.8	-1.8219	0.0731	
30–34	34	7.9 (5.5)	9.2 (5.9)	1.3	-1.8566	0.0723	
35–44	33	10.9 (7.8)	12.7 (8.0)	1.8	-2.2285	0.0330	0.39
45–54	14	11.0 (6.3)	11.9 (7.6)	0.9	-0.6920	0.5313	
55–64	2	17.5 (3.5)	22.0 (9.9)	4.5	-0.4470	1.0000	
**LS**							
LA	12	9.4 (4.7)	12.3 (7.8)	2.9	-2.1290	0.0333	0.50
LF	59	7.7 (4.9)	7.4 (5.4)	-0.3	0.6861	0.4954	
LP	73	8.5 (6.8)	9.8 (6.6)	1.3	-3.1970	0.0021	0.37
LC	15	9.7 (6.8)	10.8 (8.2)	1.1	-0.8350	0.4336	
SF	13	8.7 (4.5)	7.5 (4.5)	-1.2	0.715	0.4961	

LS: living situation during lockdown. LA: living alone. LF: living with parents or other relatives. LP: living with a partner. LC: living with children aged <16 years. SF: sharing a flat with people who were not a partner or family. t: Student’s t-test. Z: PRS Wilcoxon. p: significance level. d: Cohen’s d. Diff.: absolute difference between before and during lockdown.

Considering that only people aged 18–24 years (Table 1) and those who lived with parents or other relatives (Table 4) showed a significant decrease in daily cigarette consumption during the lockdown, four groups were created based on their family situation before and during lockdown: 1) neither before nor during did they live with their parents/relatives (n=21); 2) both before and while they lived with their parents/relatives (n=24); 3) before the pandemic they did not live with their parents/relatives and during lockdown they began to live with them (n=17); and 4) before the pandemic they lived with their parents/relatives and during lockdown they stopped doing so (n=1).

For smokers aged 18–24 years who did not live with their parents/relatives neither before nor during lockdown, their average daily cigarette consumption did not change (4.05 before; 3.48 during; Z=0.232, p=0.8412). Among those who reported living with their parents or other relatives before and during the lockdown, a significant decrease in their average consumption was observed (6.04 before; 4.13 during; Z=2.597, p<0.05). Among those who reported that before the pandemic, they did not live with their parents or other relatives, and during lockdown, they did so, their average consumption also decreased significantly (5.65 before; 3.82 during; Z=2.144, p<0.05).

## DISCUSSION

The present study adds to the knowledge of tobacco consumption among the university students, both before the pandemic and during the COVID-19 lockdown. Unlike other studies, it reports novel findings as it includes university students aged 18–64 years. Furthermore, it allows identifying vulnerable groups susceptible to increasing their tobacco consumption, which places them at greater risk of addiction and suffering from other negative impacts on their health.

At the epidemiological level, almost 2 in 10 students reported consuming tobacco in the six months prior to the lockdown, or during it. Before the pandemic, a consumption frequency of 20 days or more predominated (67.7%); and daily consumption in the last week was reported by 63.4% of the participants for the lockdown period. Cigarette consumption in this last group increased significantly during the lockdown in women but not in men; and, depending on age, only in those aged 35–44 years. The increase in consumption in these groups of university students was a risk factor for first order diseases and for developing an addictive disorder.

In the present study, no significant differences were found between the periods before and during the lockdown in terms of cigarette consumption or ENDS for the sample of participating university students or according to gender. This finding is in contrast to what was reported in another study carried out in the same period in Spain^24^, in which an increase in the amount consumed during lockdown was observed. However, a significant reduction in cigarette consumption was found in the youngest group (aged 18–24 years). The EDADES-COVID19 survey also observed a reduction in the consumption prevalence in this age group, although it did not analyze changes in the number of cigarettes consumed^25^. To interpret this finding in the context of previous literature, we must consider the broad age range used in the sample of this study compared to other studies that use university samples aged 18–30 years. Considering the subsamples in our study that correspond to this range, the average cigarette consumption reduction is in line with previous findings^13^.

Furthermore, for the subsamples corresponding to people aged 30–64 years, tobacco consumption remained at similar levels to those before the start of the pandemic, which is in line with studies carried out with the general population^26–28^. In any case, it must be considered that these statistically significant decreases or increases in cigarette consumption before the pandemic and during the lockdown translate into average variations of approximately one to two cigarettes. From a clinical perspective, the relevance of these differences can be considered small. However, an increase in the average consumption of cigarettes determines a moment of greater risk of consumption, and therefore the need to intensify interventions with this population. At the same time, a decrease in consumption signals a favorable moment to encourage smoking cessation.

In addition, an analysis was carried out in the present study to determine which population groups had decreased, maintained, or increased their cigarette consumption. The first observation is that around 4 in 10 consumers decreased their consumption in women and men. However, a similar proportion increased their consumption, with the increase slightly higher in women than in men. Considering gender and age, women aged >24 years, especially aged 35–44 years and 55–64 years, and men aged >30 years, especially those aged 45–54 years, are identified as the groups most vulnerable to increasing their tobacco consumption. These findings suggest two reflections. First, regarding the reasons behind the most significant decrease in average cigarette consumption of the youngest group of university students (aged 18–24 years), we agree with other authors^13^ that it is probably due to the closure of campuses and students not being able to get together and engage in social events due to the social isolation measures put in place to contain the pandemic^12^. However, why has this same phenomenon not been observed among older university students? It seems evident that other factors must be analyzed.

In this sense, the present study incorporated the variable of the living situation during the lockdown, given that this circumstance can help to explain better some of the changes observed in tobacco consumption. In this regard, significant increases were observed in those living alone, or with a partner; by contrast, consumption decreased significantly in those living with their parents or other relatives. During the lockdown period, cigarette consumption among those who were living alone was observed to be considerably lower than among those who were living with a partner or with under-aged children. However, it was higher among those who were sharing a flat. Considering gender and living situation, women who live alone or with a partner, and men who live alone or with under-aged children, appear to be the groups most vulnerable to increased tobacco consumption.

In general terms, during the COVID-19 lockdown period, tobacco consumption among the university students aged 18–64 years remained at pre-pandemic levels. However, the consideration of gender, age and living situation presents a heterogeneous reality with groups at greater risk of increasing their consumption. These groups consist of women aged >24 years, especially between 35–44 years and 55–64 years, who live alone or with a partner, and men aged >30 years, especially between 45–54 years, who live alone or with under-aged children. These profiles are consistent with and complement those observed by other authors, who identify an increase in consumption among women and the older adult population^24,29^. Some studies have reported that women had a more significant increase in tobacco consumption, possibly related to stressors linked to having to combine family and work responsibilities^26^. On the other hand, other studies^30^ pointed out that the profile of those who increased their consumption included those living alone and working outside the home. Some likely causes of this increase are feeling more stressed, being alone, and visiting fewer places where smoking is prohibited^29^.

In the present study, the youngest group (18–24 years) and those who lived with their parents or other relatives were the only ones who showed a significant decrease in cigarette consumption. It is consistent to think that both findings are related, in the sense that with the interruption of face-to-face university teaching, the youngest university students returned to their family home during the lockdown period. This is corroborated in the present study, were only significant decreases in cigarette consumption among young people aged 18–24 years who lived with their parents or other relatives during the lockdown, regardless of whether they lived with them before lockdown or had another living situation. However, the cigarette consumption of young people of the same age who did not live with their parents or other relatives before or during the lockdown did not vary significantly. However, according to living situation, no differences were observed in the average daily consumption before or during lockdown.

Approximately 2 in10 university students were women who used cigarettes before the pandemic but stopped their consumption during lockdown. In regard to age, greater abstinence was observed in the younger groups (aged 18–24 years) and decreased as age increased, with none in the age group of 55–64 years reporting quitting smoking. According to the living situation during the lockdown, the highest rate of quitting was observed among those who lived with their parents or other relatives, followed by those who shared a flat, those who lived alone, lived in family units with under-aged children, and finally in those who lived with their partner. It is important to point out that none of the people who smoked cigarettes daily during the pre-pandemic period reported having stopped smoking during the lockdown.

These findings align with those reported by other investigations^30^, who found that 10% of previous consumers quit smoking during this period, especially young people, students, people who were part of temporary workforce reduction programs, and those who lived with their family.

### Limitations

Among the limitations of the study, is the convenience sample, without random selection or stratified sampling, which makes it difficult to generalize the findings. Likewise, the sample distribution in the different comparison groups by age, gender and living situation is not homogeneous. Other variables not considered may be influencing the results. Even though the limitations inherent to the lockdown restrictions justify collecting the data through an online survey, it must be regarded as a limitation in terms of how representative is the sample, given that not all the population had internet access. Although self-reports are considered valid and reliable strategies because they guarantee the anonymity of the participant and the confidentiality of the data^31^, it should be noted that self-reports of changes in tobacco consumption are subjective perceptions and may be influenced by social desirability biases by the social and cultural nuances in Spain, and by the pressure and influence of the media on these perceptions. All the variables being self-reported may lead to various biases due to under- or overestimation, or recall bias, especially when retrospectively asked about tobacco consumption during the six months before the pandemic. Given the small sample size of the dual cigarette and ENDS user subgroups, they were included in the analyses in both the cigarette user subsample and the ENDS user subsample. However, it is of interest for future studies to analyze the characteristics and possible associated variables of each of these three subgroups, with a particular focus on dual users.

## CONCLUSIONS

In view of the results of this study, tobacco consumption among the university students during the COVID-19 lockdown remained at pre-pandemic levels. The majority of young people aged 18–24 years reduced the number of cigarettes consumed or even quit smoking. However, women aged >24 years who lived alone or with a partner, and men aged >30 years who live alone or with under-aged children, were identified as vulnerable groups at greater risk of increasing their consumption. These findings seem to support the hypothesis about the decrease in tobacco consumption among younger university students and, instead, the increase among those aged >30 years, probably due to the different socio-family and contextual consumption characteristics among these groups. In addition to policies to promote tobacco-free campuses, prevention actions, early detection, and brief counselling for those who consume tobacco must be implemented. Considering the offer of face-to-face and online studies and the broad age range of those who attend different levels of university studies, it is essential to extend these measures.

## Data Availability

The data supporting this research are available from the authors on reasonable request.
